# Association of the *MLXIPL/TBL2* rs17145738 SNP and serum lipid levels in the Guangxi Mulao and Han populations

**DOI:** 10.1186/1476-511X-12-156

**Published:** 2013-10-25

**Authors:** Xiao-Na Zeng, Rui-Xing Yin, Ping Huang, Ke-Ke Huang, Jian Wu, Tao Guo, Quan-Zhen Lin, Lynn Htet Htet Aung, Jin-Zhen Wu, Yi-Ming Wang

**Affiliations:** 1 Department of Cardiology, Institute of Cardiovascular Diseases, The First Affiliated Hospital, Guangxi Medical University, 22 Shuangyong Road, Nanning 530021, Guangxi, People’s Republic of China; 2 College of Stomatology, Guangxi Medical University, Nanning 530021, Guangxi, People’s Republic of China

## Abstract

**Background:**

The rs17145738 single nucleotide polymorphism (SNP) near MLX interacting protein-like/transducin (beta)-like 2 (MLXIPL/TBL2) loci is associated with serum lipid levels, but the results are inconsistent in diverse ethnic/racial groups. The current study was to investigate the association of *MLXIPL/TBL2* rs17145738 SNP and several environmental factors with serum lipid profiles in the Guangxi Mulao and Han populations.

**Methods:**

A total of 649 subjects of Mulao nationality and 712 participants of Han nationality aged 16–84 years were randomly selected from our previous stratified randomized samples. Genotyping was performed by polymerase chain reaction and restriction fragment length polymorphism combined with gel electrophoresis, and then confirmed by direct sequencing.

**Results:**

Serum apolipoprotein (Apo) B levels were higher in Mulao than in Han (*P* < 0.001). There were no significant differences in the genotypic and allelic frequencies of the *MLXIPL/TBL2* rs17145738 SNP between the two ethnic groups or between males and females. The T allele carriers had higher triglyceride (TG) and ApoB levels in Mulao, and higher total cholesterol (TC) and low-density lipoprotein cholesterol (LDL-C) levels in Han than the T allele non-carriers (*P* < 0.05 for all). Subgroup analyses showed that the T allele carriers had higher ApoB levels in both Mulao and Han females than the T allele non-carriers, but the T allele carriers had lower ApoB levels in Han males than the T allele non-carriers (*P* < 0.05, respectively). The T allele carriers in Han had higher TC, high-density lipoprotein cholesterol (HDL-C) levels and ApoA1/ApoB ratio and lower TG levels in males, and higher LDL-C levels and lower ApoA1/ApoB ratio in females than the T allele non-carriers (*P* < 0.05 for all). Serum TC levels in the combined population of the two ethnic groups and in Han, and HDL-C levels in Han males were correlated with genotypes (*P* < 0.05 for all). Serum lipid parameters were also correlated with several environmental factors (*P* < 0.05-0.01).

**Conclusions:**

The association of *MLXIPL/TBL2* rs17145738 SNP and serum lipid profiles is different between the Mulao and Han populations. There is a sex-specific association in the both ethnic groups.

## Introduction

Compelling evidence has demonstrated that serum lipid and lipoprotein concentrations are tightly associated with coronary artery disease (CAD) [1] which is the major leading causes of death and disability worldwide [2], and targets for therapeutic intervention [1,3]. It is well-established that serum lipid profiles are modulated by genetic [4,5] and multiple environmental risk factors [6,7] and their interactions [8-10]. Twin and family studies have suggested that approximately half of the inter-individual variants in serum lipid phenotypes can be explained by genetic variants [11].

During the past few years, genome-wide association studies (GWAS) have shown great success in identified several novel variants associated with plasma lipid and lipoprotein levels [4,5,12,13], such as single nucleotide polymorphisms (SNPs) localized at the MLX interacting protein-like/transducin (beta)-like 2 (MLXIPL/TBL2) loci. *MLXIPL/TBL2* loci containing genes encoding *TBL2* and *MLXIPL* which is also called carbohydrate-responsive element binding protein (chREBP). *TBL2* is associated with triglyceride (TG) metabolism [14]. Research has showed that ectopic *TBL2* over-expression significantly reduces cellular cholesterol and miRNA-mediated knockdown raises 15-30% cholesterol levels. However the role for *TBL2* in modulation of cellular cholesterol is undefined at this time. The mechanism of *MLXIPL* in lipid metabolism is also unclear in detail, the function of *MLXIPL* as a transcriptional mediator is described to connect glycolysis with lipogenesis, and regulate lipogenesis and glucose utilization in the liver by a phosphorylation/dephosphorylation pathway. In the liver, chREBP mediates activation of several regulator enzymes of glycolysis and lipogenesis [15,16]. When the hepatocytes have ample glucose, xylulose-5-phosphate is produced in the pentose phosphate pathway and activates protein phosphatase 2A, which dephosphorylates chREBP and activated it translocated to the nucleus. Nuclear chREBP combined with MLX and binds carbohydrate response elements to channel the transcription of genes involved in glycolysis, lipogenesis, TG synthesis and very-low-density lipoprotein secretion.

The rs17145738 SNP is a C > T variation located on human chromosome 7 (http://www.ncbi.nlm.nih.gov/gene) downstream of *MLXIPL/TBL2* loci and is confirmed to be relevant to TG metabolism [4,12,17], but the results are controversial in prior studies. The inconsistent results should invigorate interest in characterize the full impact of the relationship between the rs17145738 SNP and serum lipid profiles. Furthermore, the replication of this association has not been detected in the Chinese minorities so far.

China is a multiethnic country. Other than the majority Han nationality, there are 55 ethnic minorities living in China. Most of these minorities inhabit peripheral regions of China, especially border provinces such as Guangxi, where special landforms like the Karst Mountains vastly influenced their lives and history. Mulao nationality is one of the 55 minorities with population of 207,352 (the fifth national census statistics of China in 2000). Ninety percent of them live in the Luocheng Mulao Autonomous County of Hechi, Guangxi Zhuang Autonomous Region, People’s Republic of China. A previous study has shown that the genetic relationship between Mulao nationality and other minorities in Guangxi was much closer than that between Mulao and Han or Uighur nationality [18]. To the best of our knowledge, however, the association of rs17145738 SNP and serum lipid levels has not been previously reported in this population. Therefore, the aim of the present study was to detect the association of *MLXIPL/TBL2* rs17145738 SNP and several environmental factors with serum lipid profiles in the Mulao and Han populations.

## Materials and methods

### Study population

The study included 649 unrelated subjects of Mulao nationality from Luocheng Mulao Autonomous County, Guangxi Zhuang Autonomous Region, Pepole’s Republic of China. All of the subjects were randomly selected from our previous stratified randomized samples [9,10]. The age of the subjects ranged from 16 to 84 years, mean age was 52.40 ± 15.61 years. There were 290 males (44.68%) and 359 females (55.32%). We simultaneously surveyed 712 subjects of Han nationality who reside in the same villages by the same method. The mean age of the subjects was 52.55 ± 15.24 years (range 16–84 years). There were 316 males (44.38%) and 396 females (55.62%).

All study subjects were healthy peasants, and had no evidence of diseases related to atherosclerosis, CAD and diabetes. None of them had been treated with β-adrenergic blocking agents and lipid-lowering medications such as statins or fibrates. The present study was approved by the Ethics Committee of the First Affiliated Hospital, Guangxi Medical University. All participants gave written informed consent.

### Epidemiological survey

The survey was carried out using internationally standardized methods [19]. Information on demographics, socioeconomic status and lifestyle was collected with standardized questionnaires. The alcohol information included questions about the number of liangs (about 50 g) of rice wine, corn wine, rum, beer, or liquor consumed during the preceding 12 months. Alcohol consumption was categorized into subgroups of grams of alcohol per day: < 25 and ≥ 25. Smoking status was categorized into subgroups of cigarettes per day: < 20 and ≥ 20. The physical examination included body height, body weight, and waist circumference. Sitting blood pressure was measured three times with the use of a mercury sphygmomanometer after a 5-minute of rest, and the average of the three measurements was recorded. Systolic blood pressure was determined by the first Korotkoff sound, and diastolic blood pressure by the fifth Korotkoff sound. Hypertension was diagnosed according to the criteria of 1999 World Heath Organization-International Society of Hypertension Guidelines for the management of hypertension [20]. Body weight was measured using a portable balance scale. Subjects were weighed in a minimum of clothing with shoes off. Height was measured using a stadiometer. Body mass index (BMI) was calculated as weight (kg) divided by height (m) squared. Normal weight, overweight and obesity were defined as a BMI < 24, 24–28, and > 28 kg/m^2^; respectively [21].

### Biochemical analyses

The subjects who did not fast or fasted < 12 hours were excluded before testing. Venous blood sample was drawn from a forearm vein. A part of the sample was collected into a glass tube and used to determine serum lipid levels. Another part of the sample was transferred into a tube with anti-coagulate solution (4.80 g/L citric acid, 14.70 g/L glucose, and 13.20 g/L tri-sodium citrate) and used to extract deoxyribonucleic acid (DNA). The levels of total cholesterol (TC), TG, high-density lipoprotein cholesterol (HDL-C) and low-density lipoprotein cholesterol (LDL-C) in the samples were measured using standard enzymatic methods. Serum apolipoprotein (Apo) A1 and ApoB levels were assessed by the immunoturbidimetric immunoassay. The ratio of ApoA1 to ApoB was calculated. All determinations were performed with an autoanalyzer (Type 7170A; Hita-chi Ltd, Tokyo, Japan) in the Clinical Science Experiment Center of the First Affiliated Hospital, Guangxi Medical University. The normal ranges of serum TC, TG, HDL-C, LDL-C, ApoA1, ApoB levels and the ratio of ApoA1 to ApoB in our Clinical Science Experiment Center were 3.10-5.17, 0.56-1.70, 1.16-1.42, 2.70-3.10 mmol/L, 1.20-1.60, 0.80-1.05 g/L, and 1.00-2.50; respectively. The individuals with TC > 5.17 mmol/L, and/or TG > 1.70 mmol/L were defined as hyperlipidemic [7].

### DNA preparation and genotyping

**Figure 1:**
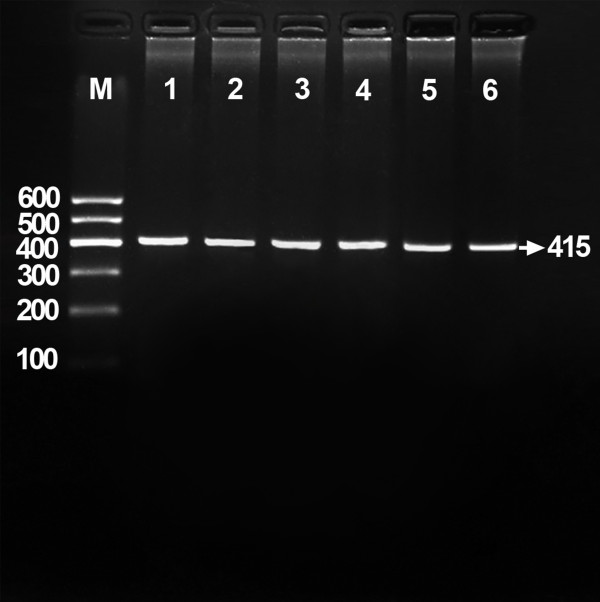
**Electrophoresis of PCR products of the samples.** Lane M: 100 bp marker ladder; lanes 1–6: samples. The 415 bp bands are the target fragments.

**Figure 2:**
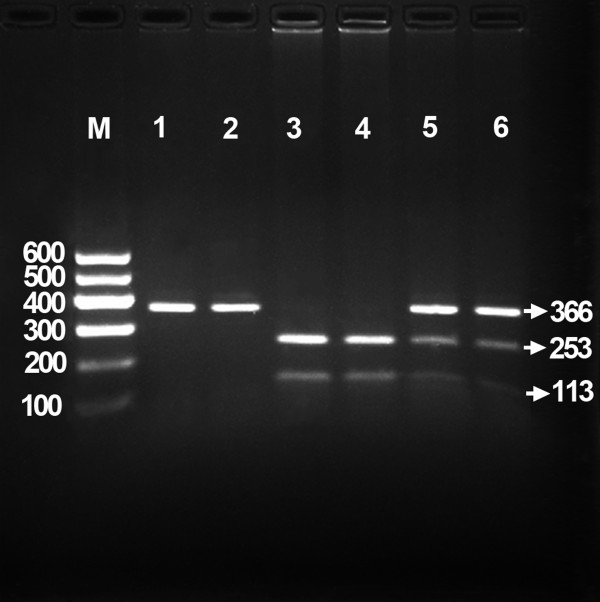
**Genotyping of the *MLXIPL/TBL2 *rs17145738 SNP.** Lane M: 100 bp marker ladder; lanes 1 and 2: TT genotype (366 bp); lanes 3 and 4: CC genotype (253- and 113-bp); and lanes 5 and 6: CT genotype (366-, 253-, and 113-bp). The 49-bp fragments were invisible in the gel owing to its fast migration speed.

**Figure 3:**
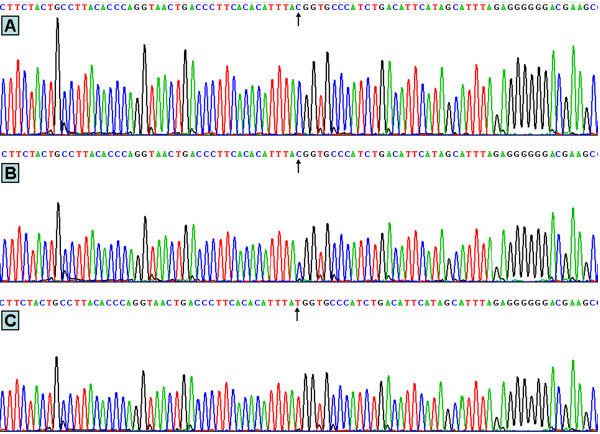
**Partial nucleotide sequences of the *MLXIPL/TBL2 *rs17145738 SNP. (A)** CC genotype, **(B)** CT genotype, and **(C)** TT genotype.

Genomic DNA was isolated from peripheral blood leukocytes using the phenol-chloroform method [7]. Genotyping of the rs17145738 SNP was performed by the polymerase chain reaction and restriction fragment length polymorphism (PCR-RFLP). The PCR amplification was performed using the following primers: F: 5'-ATGGTCCAGGAGTCTGCCC-3' and R: 5'-AGCCATCGTGCCTAGCTAAA-3' (Sangon Biotech Co., Ltd., Shanghai, People’s Republic of China). Each amplification reaction was set up in 25 μL obtained by adding: 12.5 μL 2 × *Taq* PCR MasterMix (constituent: 0.1 U *Taq* polymerase/μL, 500 μM dNTP each and PCR buffer); 1.0 μL of each primer (10 μmol/L); 2 μL genomic DNA (100 ng) and ultrapure H_2_O until reaching the final volume. The PCR condition was as follows: initial denaturation at 95°C for 5 min, followed by 32 cycles of denaturation at 95°C for 45 s, annealing at 63°C for 30 s, elongation for 45 s at 72°C and a final extension of 7 min at 72°C. Then 2 U of *Taa*I restriction enzyme, 9.0 μL nuclease-free water and 1.0 μL of 10 × buffer solution were added directly to the PCR products (5 μL) and digested at 65°C overnight, and then the fragments were separated by electrophoresis in a 2% agarose gel for 50 min at 120 V. The products of 415 bp nucleotide sequences were found in all samples (Figure 1). The PCR products have two enzyme restriction sites, one is inherently and another is the genotype identification mark. The genotypes indentified were named according to the presence or absence of the enzyme restriction sites, when a C to T transition at rs17145738 locus in the *MLXIPL-TBL2* region. The presence of the cutting site indicates the C allele, while its absence indicates the T allele (cannot be cut). Thus, the TT genotype is homozygote for the absence of the site (band at 366 bp), CT genotype is heterozygote for the presence and absence of the site (bands at 366-, 253-, and 113-bp), and CC genotype is homozygote for the presence of the site (bands at 253- and 113-bp). Because of the inherent enzyme sites, all PCR products were cut out a 49-bp fragment. The 49-bp fragment was invisible in the gel owing to its fast migration speed (Figure 2). Genotypes were scored by an experienced reader blinded to the epidemiological data and serum lipid levels. The genotypes were also confirmed randomly by DNA sequencing (Figure 3, Sangon Biotech Co., Ltd., Shanghai, People’s Republic of China).

### Statistical analyses

All statistical analyses were performed using SPSS 16.0 (SPSS Inc., Chicago, IL, USA). Quantitative variables were represented as mean ± standard deviation (serum TG levels were represented as medians and inter-quartile ranges). Qualitative variables were expressed as percentages. Comparisons of mean values of general characteristics between Mulao and Han were performed with the Student’s unpaired *t*-test. Allelic and genotypic frequencies were determined via direct counting, and the standard goodness-of-fit test was used to test the Hardy-Weinberg equilibrium. Difference in genotype distribution between the groups was tested by the Chi-square test. The association of genotypes and serum lipid parameters was tested by analysis of covariance (ANCOVA). Sex, age, BMI, blood pressure, alcohol consumption, and cigarette smoking were adjusted for the statistical analysis. In order to evaluate the association of serum lipid levels and genotypes (CC = 1, CT/TT = 2) or several environmental factors, multivariable linear regression analysis with stepwise modeling was also performed in the combined population of Mulao and Han, Mulao, Han, males, and females; respectively. A *P* value (two-tailed) of less than 0.05 was considered statistically significant.

## Results

### General characteristics and serum lipid levels

The general characteristics and serum lipid levels between the Mulao and Han populations are presented in Table 1. The levels of BMI and diastolic blood pressure were lower but the levels of ApoB, body height and the percentages of subjects who consumed alcohol were higher in Mulao than in Han (*P* < 0.05-0.001). There were no differences in the levels of age, body weight, waist circumference, systolic blood pressure, serum TC, TG, HDL-C, LDL-C, ApoA1, and the ratio of ApoA1 to ApoB or male to female, the percentages of subjects who smoked cigarettes between the two ethnic groups (*P* > 0.05 for all).

**Table 1:** Comparison of demographic, lifestyle characteristics and serum lipid levers between the Mulao and Han populations

**Parameter**	**Mulao**	**Han**	***t *(*x***^***2***^**)**	***P***
Number	649	712	-	-
Male/Female	290/359	316/396	0.013	0.911
Age (years)	52.40 ± 15.61	52.55 ± 15.24	−0.172	0.863
Height (cm)	155.21 ± 8.05	154.23 ± 8.20	2.236	0.025
Weight (kg)	52.81 ± 9.05	53.17 ± 8.90	−0.745	0.456
Body mass index (kg/m^2^)	21.87 ± 3.02	22.36 ± 3.55	−2.734	0.006
Waist circumference (cm)	75.15 ± 8.49	75.09 ± 7.34	0.135	0.893
Systolic blood pressure (mmHg)	129.63 ± 21.76	130.21 ± 19.23	−0.521	0.603
Diastolic blood pressure (mmHg)	81.05 ± 11.57	82.79 ± 11.20	−2.825	0.005
Pulse pressure (mmHg)	48.59 ± 16.45	47.42 ± 14.87	1.369	0.171
Cigarette smoking [n (%)]				
Nonsmoker	482 (74.3)	502 (70.5)		
< 20 cigarettes/day	145 (22.3)	185 (26.0)	2.536	0.281
≥ 20 cigarettes/day	22 (3.4)	25 (3.5)		
Alcohol consumption [n (%)]				
Nondrinker	493 (76.0)	555 (77.9)		
< 25 g/day	55 (8.5)	76 (10.6)	6.329	0.042
≥ 25 g/day	101 (15.5)	81 (11.4)		
Blood glucose	6.01 ± 1.62	6.10 ± 1.64	−1.034	0.301
Total cholesterol (mmol/L)	5.07 ± 1.35	5.02 ± 1.01	0.688	0.492
Triglyceride (mmol/L)	1.08 (0.80-1.60)	1.06 (0.80-1.64)	−0.602	0.547
HDL-C (mmol/L)	1.76 ± 0.47	1.74 ± 0.53	0.735	0.462
LDL-C (mmol/L)	2.93 ± 0.70	2.89 ± 0.82	0.767	0.443
Apolipoprotein (Apo) Al (g/L)	1.33 ± 0.40	1.34 ± 0.25	−0.808	0.419
ApoB (g/L)	0.97 ± 0.54	0.86 ± 0.19	5.051	0.000
ApoA1/ApoB	1.62 ± 1.03	1.64 ± 0.46	−0.381	0.703

HDL-C, high-density lipoprotein cholesterol; LDL-C, low-density lipoprotein cholesterol. The value of triglyceride was presented as median (interquartile range), the difference between the two ethnic groups was determined by the Wilcoxon-Mann–Whitney test.

### Genotypic and allelic frequencies

Table 2 shows the genotypic and allelic frequencies of *MLXIPL/TBL2* rs17145738 SNP. The genotype distribution was in Hardy-Weinberg equilibrium. The frequencies of C and T alleles were 92.99% and 7.01% in Mulao, and 93.40% and 6.60% in Han (*P* > 0.05); respectively. The frequencies of CC, CT and TT genotypes were 86.10%, 13.70% and 0.20% in Mulao, and 87.50%, 11.80% and 0.70% in Han (*P* > 0.05); respectively. The genotypic and allelic frequencies were also not different between males and females in the both ethnic groups (*P* > 0.05).

**Table 2:** Comparison of the genotypic and allelic frequencies of the rs17145738 SNP between the Mulao and Han populations [n (%)]

**Group**	**n**	**Genotype**			**Allele**	
		**CC**	**CT**	**TT**	**C**	**T**
Mulao	649	559 (86.10)	89 (13.70)	1 (0.20)	1207 (92.99)	91 (7.01)
Han	712	623 (87.50)	84 (11.80)	5 (0.70)	1330 (93.40)	94 (6.60)
*x*^*2*^	-	3.367			0.180	
*P*	-	0.186			0.671	
Mulao						
Male	290	246 (84.80)	44 (15.20)	0 (0)	536 (92.41)	44 (7.59)
Female	359	313 (87.20)	45 (12.50)	1 (0.30)	671 (93.45)	47 (6.55)
*x*^*2*^	-	1.725			0.533	
*P*	-	0.422			0.466	
Han						
Male	316	270 (85.40)	44 (14.00)	2 (0.60)	584 (92.41)	48 (7.59)
Female	396	353 (89.10)	40 (10.10)	3 (0.80)	746 (94.19)	46 (5.81)
*x*^*2*^	-	2.491			1.820	
*P*	-	0.288			0.177	

### Genotypes and serum lipid levels

As shown in Table 3, the levels of TG and ApoB were different between the CC and CT/TT genotypes in Mulao (*P* < 0.05 for each) but not in Han, the T allele carriers had higher TG and ApoB levels than the T allele non-carriers. The levels of TC and LDL-C in Han but not in Mulao were different between the CC and CT/TT genotypes (*P* < 0.05 for each), the T allele carriers had higher TC and LDL-C levels than the T allele non-carriers.

**Table 3:** Comparison of the genotypes and serum lipid levels between the Mulao and Han populations

**Ethnic group/Genotype**	**n**	**TC (mmol/L)**	**TG (mmol/L)**	**HDL-C (mmol/L)**	**LDL-C (mmol/L)**	**ApoA1 (g/L)**	**ApoB (g/L)**	**ApoA1/ApoB**
Mulao								
CC	559	5.06 ± 1.38	1.07 (0.76−1.57)	1.76 ± 0.48	2.92 ± 0.91	1.32 ± 0.41	0.95 ± 0.52	1.64 ± 1.09
CT/TT	90	5.14 ± 1.11	1.23 (0.84−1.72)	1.72 ± 0.42	2.97 ± 0.81	1.36 ± 0.33	1.07 ± 0.66	1.52 ± 0.59
*F*	-	0.431	−1.993	0.982	0.876	1.249	4.015	1.391
*P*	-	0.512	0.046	0.322	0.350	0.264	0.046	0.239
Han								
CC	623	4.99 ± 0.98	1.07 (0.82−1.65)	1.73 ± 0.54	2.88 ± 0.83	1.34 ± 0.26	0.86 ± 0.20	1.64 ± 0.48
CT/TT	89	5.27 ± 1.14	0.98 (0.74−1.45)	1.78 ± 0.40	3.01 ± 0.76	1.36 ± 0.21	0.86 ± 0.16	1.63 ± 0.34
*F*	-	8.065	−1.323	1.097	5.018	0.636	1.552	1.741
*P*	-	0.005	0.186	0.295	0.025	0.425	0.213	0.187
Mulao/Male								
CC	246	5.16 ± 1.60	1.15 (0.82−1.92)	1.74 ± 0.53	2.87 ± 0.92	1.32 ± 0.44	1.03 ± 0.62	1.52 ± 0.72
CT/TT	44	5.14 ± 1.22	1.25 (0.84−2.49)	1.71 ± 0.42	2.84 ± 0.64	1.36 ± 0.38	1.10 ± 0.72	1.48 ± 0.58
*F*	-	0.193	−1.161	0.151	0.099	1.019	0.656	0.384
*P*	-	0.661	0.245	0.698	0.754	0.314	0.419	0.536
Mulao/Female								
CC	313	4.98 ± 1.18	1.00 (0.75−1.40)	1.78 ± 0.43	2.96 ± 0.91	1.32 ± 0.40	0.90 ± 0.41	1.73 ± 1.29
CT/TT	46	5.13 ± 1.00	1.12 (0.85−1.47)	1.73 ± 0.39	3.10 ± 0.93	1.36 ± 0.27	1.03 ± 0.59	1.57 ± 0.60
*F*	-	1.168	−1.619	1.102	1.787	0.444	4.483	1.047
*P*	-	0.280	0.105	0.294	0.182	0.506	0.035	0.307
Han/Male								
CC	270	5.13 ± 0.98	1.22 (0.92−1.85)	1.65 ± 0.40	2.95 ± 0.82	1.34 ± 0.27	0.91 ± 0.20	1.53 ± 0.46
CT/TT	46	5.43 ± 1.42	0.90 (0.73−1.51)	1.79 ± 0.39	2.95 ± 0.74	1.40 ± 0.23	0.87 ± 0.17	1.66 ± 0.33
*F*	-	5.895	−2.930	4.511	1.052	2.494	5.043	4.474
*P*	-	0.016	0.003	0.034	0.306	0.115	0.025	0.035
Han/Female								
CC	353	4.89 ± 0.97	0.97 (0.74−1.53)	1.79 ± 0.63	2.82 ± 0.83	1.34 ± 0.24	0.81 ± 0.19	1.73 ± 0.48
CT/TT	43	5.09 ± 0.73	1.20 (0.84−1.36)	1.78 ± 0.41	3.07 ± 0.78	1.32 ± 0.19	0.85 ± 0.15	1.60 ± 0.34
*F*	-	2.878	0.941	0.320	4.892	1.252	4.653	6.316
*P*	-	0.091	0.347	0.572	0.028	0.264	0.032	0.012

TC, total cholesterol; TG, triglyceride; HDL-C, high-density lipoprotein cholesterol; LDL-C, low-density lipoprotein cholesterol; ApoA1, apolipoprotein A1; ApoB, apolipoprotein B; ApoA1/ApoB, the ratio of apolipoprotein A1 to apolipoprotein B. The association of genotypes and serum lipid parameters was tested by analysis of covariance (co-variables include sex, age, BMI, blood pressure, alcohol consumption, and cigarette smoking). The value of TG was presented as median (interquartile range), the difference between the genotypes was determined by the Wilcoxon-Mann–Whitney test.

Subgroup analyses according to gender showed that the levels of ApoB in Mulao females but not in males were different between the CC and CT/TT genotypes (*P* < 0.05), the T allele carriers had higher ApoB levels than the T allele non-carriers. The levels of TC, TG, HDL-C, ApoB, and the ratio of ApoA1 to ApoB in Han males were different between the CC and CT/TT genotypes (*P* < 0.05-0.01), the T allele carriers had higher TC, HDL-C levels and the ApoA1/ApoB ratio, and lower TG and ApoB levels than the T allele non-carriers. The levels of LDL-C, ApoB, and the ratio of ApoA1 to ApoB in Han females were also different between the CC and CT/TT genotypes (*P* < 0.05 for all), the T allele carriers had higher LDL-C and ApoB levels and lower ApoA1/ApoB ratio than the T allele non-carriers.

### Risk factors for serum lipid parameters

Multiple linear regression analysis showed that serum TC levels were correlated with genotypes in combined population of Mulao and Han (*P* < 0.05), and in Han (*P* < 0.05) but not in Mulao (Table 4).

**Table 4:** Correlation between serum lipid parameters and genotypes in Mulao and Han populations

**Lipid**	**Relative factor**	**Unstandardized coefficient**	**Std. error**	**Standardized coefficient**	***t***	***P***
Mulao plus Han						
TC	Genotype	0.179	0.091	0.051	1.978	0.049
Han						
TC	Genotype	0.269	0.107	0.088	2.521	0.012
Han/male						
HDL-C	Genotype	0.122	0.061	0.108	2.006	0.046

TC, total cholesterol; TG, triglyceride; HDL-C, high-density lipoprotein cholesterol. The correlation was determined by multivariable linear regression analysis with stepwise modeling.

When serum lipid data were analyzed according to gender, the levels of HDL-C in Han were associated with genotypes in males but not in females (*P* < 0.05, Table 4).

We also found that serum lipid parameters were correlated with several environmental factors such as age, gender, BMI, alcohol consumption, cigarette smoking, blood pressure and blood glucose in both ethnic groups (*P* < 0.05-0.001, Tables 5 and 6).

**Table 5:** Correlation between serum lipid parameters and environmental risk factors in the Mulao and Han populations

**Lipid parameter**	**Risk factor**	**Unstandardized coefficient**	**Std. error**	**Standardized coefficient**	***t***	***P***
Mulao plus Han						
TC	Waist circumference	0.013	0.005	0.091	2.591	0.010
	Age	0.011	0.002	0.138	5.122	0.000
	Alcohol consumption	0.211	0.044	0.126	4.759	0.000
	Body mass index	0.034	0.012	0.095	2.729	0.006
	Diastolic blood pressure	0.007	0.003	0.071	2.564	0.010
TG	Waist circumference	0.063	0.007	0.235	8.961	0.000
	Alcohol consumption	0.523	0.081	0.169	6.498	0.000
	Blood glucose	0.085	0.035	0.063	2.449	0.014
HDL-C	Waist circumference	−0.010	0.002	−0.161	−5.838	0.000
	Alcohol consumption	0.117	0.023	0.166	5.214	0.000
	Gender	0.127	0.032	0.126	2.958	0.000
	Age	0.003	0.001	0.087	2.957	0.003
	Systolic blood pressure	−0.002	0.001	−0.064	−2.121	0.034
LDL-C	Body mass index	0.049	0.007	0.187	7.152	0.000
	Age	0.010	0.001	0.179	6.837	0.000
	Cigarette smoking	−0.096	0.042	−0.060	−2.279	0.023
ApoA1	Alcohol consumption	0.142	0.015	0.302	9.640	0.000
	Gender	0.104	0.021	0.155	4.947	0.000
	Age	0.001	0.001	0.058	2.215	0.027
ApoB	Waist circumference	0.007	0.002	0.141	3.963	0.000
	Nation	−0.120	0.021	−0.148	−5.689	0.000
	Gender	−0.074	0.022	−0.091	−3.412	0.000
	Blood glucose	0.022	0.006	0.089	3.373	0.004
	Body mass index	0.010	0.004	0.082	2.360	0.011
ApoA1/ApoB	Waist circumference	−0.009	0.004	−0.094	−2.594	0.010
	Blood glucose	−0.038	0.013	−0.078	−2.906	0.004
	Gender	0.212	0.050	0.134	4.204	0.000
	Alcohol consumption	0.112	0.035	0.100	3.190	0.001
	Body mass index	−0.025	0.008	−0.105	−2.984	0.003
Mulao						
TC	Body mass index	0.075	0.017	0.169	4.395	0.000
	Age	0.011	0.003	0.123	3.204	0.001
	Alcohol consumption	0.194	0.070	0.107	2.791	0.005
TG	Waist circumference	0.061	0.011	0.212	5.596	0.000
	Alcohol consumption	0.598	0.124	0.183	4.830	0.000
HDL-C	Body mass index	−0.036	0.006	−0.230	−6.053	0.000
	Alcohol consumption	0.111	0.029	0.175	3.793	0.000
	Gender	0.120	0.044	0.127	2.755	0.006
LDL-C	Body mass index	0.053	0.011	0.177	4.625	0.000
	Age	0.008	0.002	0.137	3.577	0.000
	Alcohol consumption	−0.132	0.046	−0.109	−2.846	0.005
ApoA1	Alcohol consumption	0.146	0.025	0.268	5.766	0.000
	Gender	0.122	0.038	0.150	3.226	0.001
	Pulse pressure	0.002	0.001	0.086	2.252	0.025
ApoB	Waist circumference	0.013	0.002	0.198	5.143	0.000
ApoA1/ApoB	Waist circumference	−0.018	0.005	−0.144	−3.692	0.000
	Blood glucose	−0.051	0.025	−0.080	−2.051	0.041
Han						
TC	Diastolic blood pressure	0.016	0.003	0.178	4.783	0.000
	Alcohol consumption	0.253	0.054	0.168	4.699	0.000
	Age	0.010	0.002	0.153	4.159	0.000
	Body mass index	0.043	0.010	0.150	4.143	0.000
TG	Waist circumference	0.058	0.009	0.231	6.404	0.000
	Cigarette smoking	0.638	0.124	0.178	5.140	0.000
	Blood glucose	0.218	0.043	0.184	5.086	0.000
	Diastolic blood pressure	0.029	0.006	0.167	4.559	0.000
	Age	−0.017	0.005	−0.130	−3.514	0.000
HDL-C	Waist circumference	−0.009	0.003	−0.137	−3.629	0.000
	Gender	0.158	0.046	0.148	3.416	0.001
	Alcohol consumption	0.105	0.034	0.134	3.099	0.002
LDL-C	Age	0.012	0.002	0.216	6.053	0.000
	Body mass index	0.047	0.008	0.203	5.620	0.000
	Cigarette smoking	−0.267	0.068	−0.176	−3.916	0.000
	Gender	−0.230	0.075	−0.140	−3.086	0.002
ApoA1	Alcohol consumption	0.128	0.016	0.340	7.839	0.000
	Gender	0.107	0.024	0.211	4.478	0.000
	Body mass index	−0.007	0.003	−0.097	−2.695	0.007
	Cigarette smoking	0.056	0.022	0.119	2.558	0.011
ApoB	Waist circumference	0.004	0.001	0.179	4.262	0.000
	Alcohol consumption	0.054	0.010	0.187	5.631	0.000
	Blood glucose	0.020	0.004	0.167	5.018	0.000
	Body mass index	0.011	0.002	0.195	4.746	0.000
	Diastolic blood pressure	−0.002	0.001	−0.099	−2.314	0.021
ApoA1/ApoB	Body mass index	−0.024	0.006	−0.186	−4.256	0.000
	Diastolic blood pressure	−0.003	0.001	−0.132	−3.733	0.000
	Waist circumference	−0.008	0.003	−0.134	−3.045	0.002
	Gender	0.239	0.043	0.256	5.521	0.000
	Cigarette smoking	0.151	0.039	0.176	3.886	0.000
	Alcohol consumption	0.071	0.029	0.103	2.436	0.015

TC, total cholesterol; TG, triglyceride; HDL-C, high-density lipoprotein cholesterol; LDL-C, low-density lipoprotein cholesterol; ApoA1, apolipoprotein A1; ApoB, apolipoprotein B; ApoA1/Apo B, the ratio of apolipoprotein A1 to apolipoprotein B. The correlation was determined by multivariable linear regression analysis with stepwise modeling.

**Table 6:** Correlative factors for serum lipid parameters between males and females in both ethnic groups

**Lipid parameter**	**Relative factor**	**Unstandardized coefficient**	**Std. error**	**Standardized coefficient**	***t***	***P***
Mulao / Male						
TC	Body mass index	0.103	0.030	0.197	3.418	0.001
	Alcohol consumption	0.201	0.100	0.116	2.018	0.045
TG	Waist circumference	0.097	0.022	0.250	4.419	0.000
	Alcohol consumption	0.516	0.215	0.136	2.400	0.017
HDL-C	Waist circumference	−0.013	0.003	−0.227	−4.022	0.000
	Alcohol consumption	0.112	0.033	0.194	3.428	0.001
LDL-C	Body mass index	0.046	0.017	0.154	2.651	0.008
	Alcohol consumption	−0.126	0.057	−0.128	−0.128	0.028
ApoA1	Alcohol consumption	0.141	0.027	0.293	5.279	0.000
	Pulse pressure	0.004	0.002	0.144	2.589	0.010
	Blood glucose	−0.029	0.012	−0.129	−2.320	0.021
ApoB	Waist circumference	0.012	0.004	0.172	2.957	0.003
ApoA1/ApoB	Waist circumference	−0.019	0.005	−0.235	−4.158	0.000
	Alcohol consumption	0.141	0.044	0.179	3.197	0.002
	Blood glucose	−0.042	0.021	−0.115	−2.034	0.043
Mulao / Female						
TC	Age	0.015	0.004	0.196	3.803	0.000
	Body mass index	0.053	0.019	0.139	2.695	0.007
TG	Alcohol consumption	1.434	0.306	0.237	4.683	0.000
	Waist circumference	0.024	0.006	0.189	3.728	0.000
HDL-C	Body mass index	−0.032	0.007	−0.230	−4.459	0.000
LDL-C	Age	0.013	0.003	0.228	4.500	0.000
	Body mass index	0.056	0.015	0.187	3.682	0.000
ApoB	Cigarette smoking	1.217	0.302	0.205	4.035	0.000
	Waist circumference	0.010	0.003	0.182	3.581	0.000
	Age	0.003	0.001	0.118	2.321	0.021
ApoA1/ApoB	Age	−0.010	0.004	−0.133	−2.526	0.012
Han / Male						
TC	Diastolic blood pressure	0.028	0.005	0.305	5.788	0.000
	Alcohol consumption	0.242	0.067	0.191	3.637	0.000
TG	Waist circumference	0.065	0.018	0.200	3.653	0.000
	Cigarette smoking	0.903	0.223	0.216	4.047	0.000
	Blood glucose	0.341	0.080	0.237	4.239	0.000
	Diastolic blood pressure	0.045	0.012	0.203	3.708	0.000
	Age	−0.019	0.009	−0.121	−2.185	0.030
HDL-C	Waist circumference	−0.012	0.003	−0.231	−4.236	0.000
	Alcohol consumption	0.096	0.026	0.201	3.673	0.000
LDL-C	Cigarette smoking	−0.281	0.072	−0.212	−3.926	0.000
	Body mass index	0.041	0.011	0.205	3.803	0.000
ApoA1	Alcohol consumption	0.127	0.017	0.395	7.437	0.000
	Body mass index	−0.008	0.003	−0.120	−2.338	0.020
	Cigarette smoking	0.051	0.023	0.114	2.161	0.031
ApoB	Waist circumference	0.004	0.001	0.154	2.716	0.007
	Diastolic blood pressure	0.004	0.001	0.226	4.559	0.000
	Alcohol consumption	0.048	0.011	0.208	4.289	0.000
	Body mass index	0.010	0.003	0.208	3.744	0.000
	Blood glucose	0.018	0.005	0.167	3.443	0.001
ApoA1/ApoB	Body mass index	−0.027	0.006	−0.251	−4.215	0.000
	Cigarette smoking	0.110	0.039	0.152	2.838	0.005
	Waist circumference	−0.010	0.003	−0.174	−2.909	0.004
	Alcohol consumption	0.071	0.029	0.134	2.474	0.014
Han/Female						
TC	Age	0.021	0.003	0.329	7.023	0.000
	Body mass index	0.065	0.015	0.205	4.378	0.000
TG	Waist circumference	0.050	0.008	0.301	6.277	0.000
	Diastolic blood pressure	0.017	0.006	0.156	3.072	0.002
	Blood glucose	0.103	0.039	0.130	2.666	0.008
	Age	−0.009	0.004	−0.109	−2.115	0.035
HDL-C	Systolic blood pressure	−0.006	0.002	−0.188	−3.282	0.001
	Age	0.007	0.002	0.171	2.983	0.003
LDL-C	Age	0.019	0.003	0.337	6.977	0.000
	Waist circumference	0.020	0.005	0.176	3.756	0.000
	Cigarette smoking	−0.601	0.216	−0.135	−2.785	0.006
ApoA1	Alcohol consumption	0.185	0.064	0.145	2.903	0.004
ApoB	Waist circumference	0.003	0.002	0.116	1.725	0.085
	Pulse pressure	0.001	0.001	0.110	2.187	0.029
	Blood glucose	0.017	0.006	0.145	3.065	0.002
	Body mass index	0.015	0.004	0.254	3.759	0.000
	Cigarette smoking	−0.167	0.046	−0.169	−3.600	0.000
	Age	0.002	0.001	0.167	3.166	0.002
ApoA1/ApoB	Systolic blood pressure	−0.006	0.001	−0.230	−4.807	0.000
	Cigarette smoking	0.616	0.117	0.243	5.261	0.000
	Body mass index	−0.035	0.007	−0.225	−4.853	0.000
	Blood glucose	−0.029	0.015	−0.093	−1.973	0.049

TC, total cholesterol; TG, triglyceride; HDL-C, high-density lipoprotein cholesterol; LDL-C, low-density lipoprotein cholesterol; ApoA1, apolipoprotein A1; ApoB, apolipoprotein B; ApoA1/ApoB, the ratio of apolipoprotein A1 to apolipoprotein B. The correlative factors were determined by multivariable linear regression analysis with stepwise modeling.

## Discussion

In the present study, we found that the levels of ApoB were higher in Mulao than in Han (*P* < 0.001). There were no significant differences in the levels of TC, TG, HDL-C, LDL-C, ApoA1 and the ratio of ApoA1 to ApoB between the two ethnic groups. These findings are somewhat inconsistent with our previous studies [9,10]. We previously showed that both LDL-C and ApoB levels were higher in Mulao than in Han [9], or the levels of ApoA1 were lower and the levels of ApoB were higher in Mulao than in Han [10]. These discrepancies may be owing to the different sampling from our previous stratified randomized samples. It is universally recognized that the concentration of lipids is influenced by environmental factors [7] as well as genetic factors [9] and their interactions [8]. The marriages of Mulao nationality are traditionally arranged by elders when children were 12 or 13 years’ old. Unlike some conservative ethnic groups, there is a preference for marriage to mother’s brother’s daughter. Therefore, we believe that the intra-ethnic marriage customs and unique traditions have made the Mulao to be a genetic characteristics distinctively nationality. The genetic background and some lipid-related genes in Mulao may be different from those in Han nationality.

The genotypic and allelic frequencies of *MLXIPL/TBL2* rs17145738 SNP in diverse racial/ethnic groups are slightly different. The frequency of T allele was 12% in European Americans, 9% in African Americans, 8% in American Indians, 7% in Mexican Americans and Hispanics [13], and 13% in the Northern Swedish population [22]. The frequency of T allele was 12% in Malay population [23], which was similar to the InChianti sample of Italy [24]. Hegele *et al.*[25] reported that the frequency of T allele was 5.1-11% in classical Fredrickson hyperlipoproteinemic patients and 14.9% in normolipidemic controls. Wang *et al.*[25] showed that the frequency of T allele was 6.8% in hypertriglyceridemic patients and 13.1% in normal controls [26]. In the present study, we showed that the frequency of T allele was 7% in Mulao and 6.6% in Han, which was closer to that in Mexican Americans and Hispanics [13] and the hypertriglyceridemic patients [26], but was lower than that in a multiethnic SHARE sample from Canada (the minor allele frequency was 8% in South Asian, 11% in Chinese, and 19% in European Caucasian; respectively) [12]. There were no significant differences in the genotypic and allelic frequencies between males and females in both ethnic groups. These findings were similar to the results of a previous study [27], Polgár *et al.*[27] found that the allelic frequency of *MLXIPL/TBL2* rs17145738 SNP in patients with stroke did not significantly differ from that in control group. These results indicate that the genetic variation of *MLXIPL/TBL2* may be similar in the Mulao and Han populations.

The relationship between rs17145738 SNP and plasma or serum lipid levels in previous GWAS is inconsistent in multiethnic populations. Several GWAS showed that the rs17145738 SNP contributed to elevated TG [4,13,28,29] and HDL-C [4,13,28,29] concentrations. Kathiresan *et al.*[13] found that the T allele was associated with higher HDL-C and lower TG concentrations in European Ancestry. These findings are partly consistent with our results. We found that the T allele carriers in Han males had higher HDL-C and lower TG levels. Moreover, Liu *et al.*[28] also reported that the rs17145738 SNP was associated with TG (TG concentration increase of 0.17 mmol/L per C allele) and HDL-C (HDL-C concentration increase of 0.04 mmol/L per T allele) in a Chinese Han population from Shanghai. In addition, Keebler *et al.*[29] showed that the T allele of rs17145738 SNP was associated with HDL-C in Non-Hispanic Whites but not in Non-Hispanic Black and Mexican Americans, and was significantly associated with TG in Non-Hispanic Whites and Mexican Americans, but no in Non-Hispanic Black. A previous study detecting the polygenic determinants of severe hypertriglyceridemia found that the rs17145738 SNP was significantly associated with severe hypertriglyceridemia [26]. However, several previous studies failed to find a significant association between the rs17145738 SNP and plasma lipid levels [23,27]. Polgár *et al.*[27] did not confirm any associations between the rs17145738 SNP and altered TG or other lipid parameters. Tai *et al*. [23] also found that the rs17145738 SNP did not show any association with lipid-related traits in Malay population. In the present study, we found that the association of *MLXIPL/TBL2* rs17145738 SNP and serum lipid levels was different between the Mulao and Han populations. The T allele carriers in Mulao had higher TG and ApoB levels, whereas the T allele carriers in Han had higher TC and LDL-C levels than the T allele non-carriers. When serum lipid parameters were analyzed according to gender, we found that the T allele carriers had higher ApoB levels in both Mulao and Han females than the T allele non-carriers, but the T allele carriers had lower ApoB levels in Han males than the T allele non-carriers. The T allele carriers in Han had higher TC, HDL-C levels and ApoA1/ApoB ratio and lower TG levels in males, and higher LDL-C levels and lower ApoA1/ApoB ratio in females than the T allele non-carriers. Multiple linear regression analysis showed that serum TC levels were correlated with genotypes in combined population of Mulao and Han, and in Han but not in Mulao. The levels of HDL-C in Han were associated with genotypes in males but not in females. These findings suggest that the rs17145738 SNP can also influence other serum lipid parameters except TG and HDL-C in our populations. However, the reason for these different findings is unclear, probably because of different study designs, sample size, sex and age structure, experimental technique and multiethnic background trait, as well as different environmental and genetic factors and gene-environmental interactions.

In the present study, we also found that the T allele carriers in Han females had higher ApoB levels and lower ApoA1/ApoB ratio, whereas the T allele carriers in Han males had lower ApoB levels and higher ApoA1/ApoB ratio than the T allele non-carriers. These opposite effects of rs17145738 SNP on serum lipid levels might exist in some populations although it was not relevant in prior studies. Thus, we hypothesize that there may be a sex-specific association of the *MLXIPL/TBL2* rs17145738 SNP and serum lipid levels in the Han population. In addition, the ratio of ApoA1 to ApoB which reflect the cholesterol balance between antiatherogenic and atherogenic lipoprotein particles has been demonstrated to have a great value in predicting cardiovascular risk [3]. Low ratio of ApoA1 to ApoB reflects the potential risk of atherosclerosis. Furthermore, the gonadal hormone is considered as a contribution factor on lipid metabolism although the reasons for sex differences in serum lipid levels are still unclear. It is generally believed that androgens induce changes in serum lipid levels that would predispose towards CAD, whereas estrogens are held to opposite effects [30]. This conclusion makes us more confusion. In theory, males have more CAD susceptibility factors than females. But in the present study the T allele is a protective factor in Han males (higher ApoA1/ApoB ratio) whereas it is an unfavorable factor for CAD in Han females (lower ApoA1/ApoB ratio). This ambivalent founding may be due to several potential limitations of this study. First, the mean age (about 52 years) of the subjects was older in the present study than in the other studies, and the gonadal hormone levels may be low. Second, the women accounted for about 55% of the subjects in the current study. Epidemiological studies have provided abundant evidence that serum lipid levels are closely related to sex. Third, the sample size is a bit small. It may not be large enough to detect the association of *MLXIPL/TBL2* rs17145738 SNP and serum lipid levels in the subgroup analyses. Finally, serum lipid levels are affected by multiple environmental, genetic factors, and their interactions. Although sex, age, BMI, blood pressure, alcohol consumption, and cigarette smoking have been adjusted for the statistical analysis, we cannot completely exclude the influence of these factors on serum lipid levels between different alleles or genotypes. To the best of our knowledge, however, the association of *MLXIPL/TBL2* rs17145738 SNP and serum ApoB levels and the ApoA1/ApoB ratio has not been previously explored. Further studies are needed to clarify.

It is well known that serum lipid concentration is highly heritable and is also modifiable by environmental factors, as well as the gene-environmental interactions [7,8]. In the present study, we also showed that serum lipid parameters were correlated with age, gender, alcohol consumption, cigarette smoking, BMI, blood pressure and blood glucose in both ethnic groups. These results suggested that the environmental factors also play an important role in determining serum lipid levels in our populations. Although Mulao and Han reside in the same region, there was significant difference in their diet and lifestyle. Rice and corn are the staple foods in both ethnic groups. Mulao people favour the sour food, they like to eat cold foods along with acidic and spicy dishes, so bean soy sauce and pickled vegetables are among their most popular dishes. They also prefer to eat animal offal which contains abundant saturated fatty acid. It has been widely accepted that high-fat diets, particularly those that contain large quantities of saturated fatty acids, increase serum cholesterol levels [6]. The American Heart Association’s recently advocate a population-wide limitation of dietary saturated fat to < 10% of calories and cholesterol < 300 mg/day to build a desirable blood cholesterol and lipoprotein profile [31]. We also found that the percentages of subjects who consumed alcohol were higher in Mulao than in Han. That may because Mulao, regardless of men and women, all favour glutinous rice wine more than Han. In September, the rice harvest, the Mulao people like to use the best rice brewed into a mellow and after-effect of great glutinous rice wine. So the Mulao nationality called The Double Ninth Festival “the wine”. The past few decades, researchers focused on the relationship between alcohol and blood lipid concentrations, because of the interest in the U shaped or J shaped between alcohol and atherogenesis [32]. Numerous studies showed that moderate alcohol intake has been associated with reduced cardiovascular events [33,34]. These results probably attributed to the changes in serum HDL-C, TG and ApoA1 levels [34,35]. However, alcohol consumption was also associated with worse hematological values of TC and LDL-C levels. Results from the Italian Longitudinal Study on Aging showed that in elderly men (65–84 years) alcohol consumption increases serum LDL-C levels [36]. A previous study of Turks also found that the levels of LDL-C, as well as ApoB and TG were increased in male drinkers, while females had decreased TG and no change in LDL-C or ApoB with alcohol [37].

## Conclusions

The present study shows that the genotypic and allelic frequencies of *MLXIPL/TBL2* rs17145738 SNP were not different between the Mulao and Han populations or between males and females in both ethnic groups. But the association of the rs17145738 SNP and serum lipid levels is different between the two ethnic groups. There is a sex-specific association of *MLXIPL/TBL2* rs17145738 SNP and serum lipid levels in the both ethnic groups.

## Competing interests

The authors declare that they have no competing interests.

## Authors’ contributions

XNZ participated in the design, undertook genotyping, and draft the manuscript. RXY conceived the study, participated in the design, carried out the epidemiological survey, collected the samples, and helped to draft the manuscript. PH, KKH, JW, TG, QZL and LHHA collaborated to the genotyping. LHHA, JZW and YMW carried out the epidemiological survey and collected the samples. All authors read and approved the final manuscript.
